# Protocol for genetic discovery and fine-mapping of multivariate latent factors from high-dimensional traits

**DOI:** 10.1016/j.xpro.2025.104198

**Published:** 2025-11-14

**Authors:** William J. Astle, Adam S. Butterworth, Jennifer L. Asimit

**Affiliations:** 1 MRC Biostatistics Unit, University of Cambridge, Cambridge CB2 0SR, UK; 2 NHS Blood and Transplant, Cambridge CB2 0PT, UK; 3 NIHR Blood and Transplant Research Unit in Donor Health and Behaviour, University of Cambridge, Cambridge CB2 0BB, UK; 4 British Heart Foundation Cardiovascular Epidemiology Unit, Department of Public Health and Primary Care, University of Cambridge, Cambridge CB2 0BB, UK; 5 Victor Phillip Dahdaleh Heart and Lung Research Institute, University of Cambridge, Cambridge CB2 0BB, UK; 6 British Heart Foundation Centre of Research Excellence, University of Cambridge, Cambridge CB2 0QQ, UK; 7 Health Data Research UK Cambridge, Wellcome Genome Campus and University of Cambridge, Cambridge CB10 1SA, UK

**Keywords:** Bioinformatics, Genetics, Genomics

## Abstract

High-dimensional traits, like blood cell traits, are often analyzed using univariate genetic analysis approaches, ignoring trait relationships. Here, we present a protocol for using the flashfmZero software for analyses of latent factors that capture variation in observed traits generated by shared underlying biological mechanisms. We describe steps for calculating genome-wide association study (GWAS) summary statistics of latent factors from GWAS of observed traits, allowing for missing trait measurements. We then describe steps for jointly fine-mapping associations from multiple latent factors.

For complete details on the use and execution of this protocol, please refer to Zhou et al.^1^

## Highlights

• Steps for using latent factor genetic analysis methods for high-dimensional traits
• Instructions for using observed trait GWAS summary statistics for latent factor analyses
• Guidance on identifying likely causal variants for high-dimensional traits

## Abstract (editor highlights)

Publisher’s note: Undertaking any experimental protocol requires adherence to local institutional guidelines for laboratory safety and ethics.

## Graphical abstract

**Figure:**
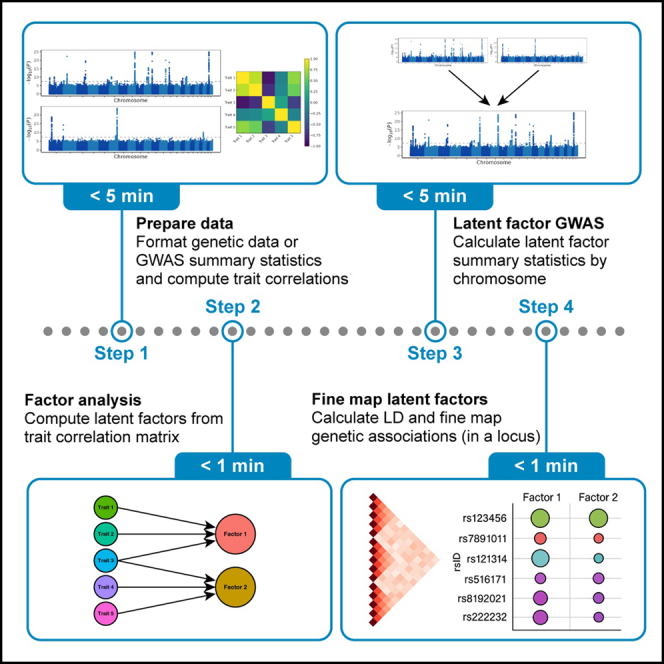

## Before you begin

In this protocol we demonstrate how to calculate latent factor genome-wide association study (GWAS) summary statistics and how to perform single- and multi-trait Bayesian fine-mapping of latent factors using the flashfmZero R package.^1^^,^^2^ We begin from GWAS summary statistics computed for each phenotype in complete blood count (CBC) reports measured using Sysmex XN analysers on the European genetic ancestry study participants in the INTERVAL BioResource.^3^^,^^4^

CBC reports are used clinically to determine properties of the peripheral blood. They generally incorporate a standard set of (‘classical’) traits, including measurements of the concentration of hemoglobin, measurements of the concentrations of the principal types of blood cell and measurements of the average volumes of red cells and platelets. In this example, we also include so-called ‘non-classical’ CBC (ncCBC) traits that do not appear in standard CBC reports but that are measured by the Sysmex XN, such as cell-type specific measurements of average cell granularity and cell nucleic acid content. Collectively, the blood traits we consider exhibit a complicated correlation structure, partly because all types of blood cell derive from a common stem cell type —the hematopoietic stem cell (HSC) —and partly because different types of mature blood cell interact, for instance, in hemostasis and in immune responses.

In the protocol, we demonstrate the derivation of latent factor GWAS summary statistics for chromosome 1 and demonstrate Bayesian fine-mapping of latent factors in a region of chromosome 1 containing *SMIM1*, a gene that expresses small integral membrane protein 1, a transmembrane protein underlying the Vel blood group.^5^^,^^6^ Variants in the *SMIM1* locus, including an expression quantitative trait locus (eQTL) of the gene and a frameshift variant responsible for the Vel blood type, have been associated with a wide variety of phenotypes relating to red blood cell biology.^3^^,^^4^^,^^7^^,^^8^^,^^9^^,^^10^^,^^11^^,^^12^

The complete set of summary statistics for GWASs of the blood cell traits performed in the INTERVAL BioResource can be obtained from the FTP sites indicated in the key resources table (or from the NHGRI-EBI GWAS Catalog). A phenotypic covariance matrix and association and linkage disequilibrium summary statistics for the *SMIM1* region computed using data from the European ancestry participants in the INTERVAL BioResource can be obtained from https://github.com/jennasimit/flashfmZero-example-data.

The R packages relied upon in the protocol are available from The R Foundation (CRAN), Bioconductor or GitHub as indicated in the key resources table.

### Innovation

Current methods for the GWAS of high-dimensional traits are often univariate, ignoring trait relationships. Associations with pleiotropic variants that are detectable with multivariate methods may be missed by univariate approaches, if each univariate association test statistic is sub-threshold. This protocol illustrates how to use flashfmZero, a zero-correlation latent factor framework that captures shared variation amongst traits. Using GWAS summary statistics from high-dimensional traits, such as blood cell or metabolic traits, the calculation of the GWAS summary statistics for latent factors underlying the traits is illustrated. The latent factor GWAS summary statistics are used to identify genetic associations, which are then fine-mapped. This gives improved precision and resolution compared to univariate fine-mapping approaches applied to the measured traits. The approach can be applied using GWAS summary statistics, it accommodates missing trait measurements, and it allows joint fine-mapping across multiple latent factors. Overall, flashfmZero represents an advancement over existing methods by leveraging trait relationships to increase power and reduce credible set sizes. It provides a parsimonious framework for interpreting genetic architecture of high-dimensional traits.

### Institutional permissions

This work involves data collected from the INTERVAL BioResource, which involves participants from the INTERVAL trial (ISRCTN24760606). The INTERVAL study received ethics committee approval from the National Research Ethics Service Committee for East of England - Cambridge East (Research Ethics Committee ref. 11/EE/0538). All participants provided informed consent.

## Key resources table

**Table:** 

REAGENT or RESOURCE	SOURCE	IDENTIFIER
**Deposited data**		

INTERVAL summary statistics for non-classical complete blood count traits	Akbari et al.^4^	http://ftp.sanger.ac.uk/pub/project/humgen/summary_statistics/sysmex_blood_cell_genetics
INTERVAL summary statistics for classical complete blood count traits	Astle et al.^3^	https://www.phpc.cam.ac.uk/ceu/haematological-traits/

**Software and algorithms**		

R 4.3.1	R Core Team^13^	https://www.r-project.org/
data.table	The R Foundation	https://cran.r-project.org/web/packages/data.table/index.html
Dplyr	The R Foundation	https://cran.r-project.org/web/packages/dplyr/index.html
reshape2	The R Foundation	https://cran.r-project.org/web/packages/reshape2/index.html
Stringr	The R Foundation	https://cran.r-project.org/web/packages/stringr/index.html
Tidyr	The R Foundation	https://cran.r-project.org/web/packages/tidyr/index.html
Readr	The R Foundation	https://cran.r-project.org/web/packages/readr/index.html
ggplot2	The R Foundation	https://cran.r-project.org/web/packages/ggplot2/index.html
RColorBrewer	The R Foundation	https://cran.r-project.org/web/packages/RColorBrewer/index.html
flashfmZero	Zhou et al.^1^	https://jennasimit.github.io/flashfmZero/jennasimit.github.io/flashfmZero/
Psych (factor analysis package)	The R Foundation	https://cran.r-project.org/web/packages/psych/index.html
BiocManager	The R Foundation	https://cran.r-project.org/web/packages/BiocManager/index.html
Rsamtools	Bioconductor	https://bioconductor.org/packages/release/bioc/html/Rsamtools.html
BSgenome.Hsapiens.UCSC.hg19	Bioconductor	https://www.bioconductor.org/packages/release/data/annotation/html/BSgenome.Hsapiens.UCSC.hg19.html
GenomicRanges	Bioconductor	https://bioconductor.org/packages/release/bioc/html/GenomicRanges.html
qctool v2	code.enkre.net; Gavin Band	https://enkre.net/cgi-bin/code/qctool/dir?ci=trunk
bigsnpR	The R Foundation	https://cran.r-project.org/web/packages/bigsnpr/index.html

**Other**		

Custom code for the INTERVAL NMR analyses that use summary statistics	Asimit^2^	https://jennasimit.github.io/flashfmZero/
Intel Xeon Platinum 8368Q processor	Intel Corporation	https://www.intel.com/content/www/us/en/products/sku/212281

## Step-by-step method details

The computation times reported were calculated using a machine with an Intel Xeon Platinum 8368Q processor (Intel Corporation).

### Input data preparation and formatting

**Timing: <5 min**

This step determines if any traits should be removed from the analysis (e.g., due to high levels of missingness). It also harmonizes the GWAS data from all traits so that the variants are aligned to the same allele in all GWASs and only variants present in all GWASs are included.

1. If a summary statistics file is unindexed, then compress it and index it using the command line tools bgzip and tabix respectively (bgzip and tabix are distributed in bcftools).

***Note:*** In this example the chromosome identifier and the 1-based physical position coordinate are stored in columns 3 and 4 of the summary statistics file respectively. All our example files use GRCh37 coordinates.

$ bgzip -c MFR_chr1.tsv > MFR_chr1.tsv.gz

$ tabix -s 3 -b 4 -e 4 MFR_chr1.tsv.gz

2. Open R and load the required packages, installing if needed.

> if (!requireNamespace("BiocManager",quietly=TRUE))

  install.packages("BiocManager")

> packages <- c("BSgenome.Hsapiens.UCSC.hg19","Rsamtools")

> for (pkg in packages) {

 if (!requireNamespace(pkg, quietly = TRUE)) {

 BiocManager::install(pkg)

}

 library(pkg, character.only=TRUE)

}

> if (!requireNamespace("remotes", quietly = TRUE)) { install.packages("remotes")

 }

> remotes::install_github("jennasimit/flashfmZero";)

> library(flashfmZero)

See troubleshooting 1 if flashfmZero cannot be installed.

3. Remove traits with an unacceptably high proportion of missing measurements (here, the missingness threshold is half of the participants, though this is somewhat arbitrary) and calculate a trait correlation matrix.
  ***Note:*** This matrix will be used later to filter traits to ensure that no three retained traits form a block of traits in which all pairwise correlations are greater than 0.99.
  a. Read in trait data, where ‘traits.csv’ is a comma separated file having traits in columns with trait names in a header row.
    > Y_start <- read.table("./traits.csv",header=TRUE,sep=",")
  b. Calculate the proportion of missing values for each trait, missing_data_fraction, by first calculating the number of non-missing values for each trait, count_non_missing_values.
    > count_non_missing_values <- unlist(as.data.frame(colSums(!is.na(Y_start))))
    > missing_data_fraction <- 1-count_non_missing_values_start/nrow(Y_start)
  c. Remove any traits that are missing measurements in more than half of the participants (though this threshold of 0.50 may be changed as desired).
    ***Note:*** The example data has a sample size of 43,059, and the maximum missingness is 32%. For smaller sample sizes (e.g. N < 20,000) a reduced missingness threshold of 0.25 is recommended for stable calculations.
    > trait_rm <- which(missing_data_fraction > 0.50)
    > if(length(trait_rm)>0) {
    > Y_start <- Y_start[,-trait_rm]
    > missing_data_fraction <- missing_data_fraction[-trait_rm]
    > }
  d. Calculate the trait correlation matrix.
    > corY_start <- cor(Y_start, use="pairwise.complete.obs", method='pearson')
4. Format summary statistics files for retained traits and store in obsgwas, a list of data frames.
  a. Select (i) an entire chromosome or (ii) a locus for analysis.
    i. Identify a region consisting of an entire chromosome.
      > region <- GRanges(seqnames = 1 , ranges = IRanges(start = 1, end = seqlengths(Hsapiens)[1]))
      ***Note:*** Here, seqlengths(Hsapiens)[1] indicates the physical length of chromosome 1.
      or.
    ii. Identify the locus of interest using the ranges argument.
      > region <- GRanges(seqnames = 1, ranges = IRanges(start = 3441528, end = 3959487))
      ***Note:*** Here, the range specified corresponds to the *SMIM1* locus.
  b. Read data for the region from each summary statistics file.
    ***Note:*** This example uses the data for the MFR (medium fluorescent percentage of reticulocytes) phenotype within the specified region from a file containing the summary statistics for chromosome 1.
    > MFR_ss_tabix <- TabixFile("./MFR_chr1.tsv.gz")
    > scanTabix(MFR_ss_tabix, param = region) %>% unlist() %>% paste(collapse="\n") %>% read_tsv(col_names=FALSE) -> MFR_ss
    > read_tsv("./MFR_chr1.tsv.gz", n_max=0) %>% colnames() -> colnames(MFR_ss)
  c. Store the regional summary statistics for the phenotype in the list of data frames.
    > obsgwas[["MFR"]]<-as.data.frame(MFR_ss)
5. Remove traits to eliminate pairwise correlations > 0.99, prioritizing traits according to the number of such correlations and the proportion of missingness.

# Build the adjacency matrix

> adj_mat <- abs(corY_start) > 0.99

> diag(adj_mat) <- FALSE # Remove self-loops

# Initialize the list of variables to trim

> variables_to_trim <- NULL

# Iteratively remove variables

> while (any(adj_mat)) {

# Compute the degree (number of low-correlation connections) for each variable

> deg <- rowSums(adj_mat)

# Compute the score: higher degree and more missing data increase the score

> score <- deg + missing_data_fraction

> remove_index<-which.max(score)

> variables_to_trim<-c(variables_to_trim, remove_index)

# Update the adjacency matrix by removing the corresponding row and column

> adj_mat[remove_index, ] = FALSE

> adj_mat[,remove_index] = FALSE

> }

6. Remove the highly correlated traits that were identified in the previous step and update the trait correlation matrix.

# Subset data frame to include only the variables to keep

> traits_to_keep<- !1:dim(Y_start)[2] %in% variables_to_trim

> Y<-Y_start[,traits_to_keep]

> write.table(as.data.frame(colnames(Y)), file="selected_phenotypes.tsv", col.names=FALSE, row.names=FALSE, quote=FALSE, sep="\t")

> corY <- corY_start[traits_to_keep, traits_to_keep]

7. Harmonise the GWAS summary statistics (remove any variants that are not present in the GWAS summary statistics for all the traits, adjust the statistics so that each variant has the same effect allele across the traits) and remove duplicate variants.
  a. Apply the harmoniseGWAS function (from the flashfmZero R package) to obsgwas, the list object containing a GWAS summary statistic data frame for each trait.
    ***Note:*** In this example, the GWAS summary statistic files are assumed to be in the format of the file for the MFR blood cell trait distributed in the GitHub repository (see key resources table), but the code is flexible to other column name formats.
    **CRITICAL:** It is assumed that all variants are on the same chromosome.
    > obsgwas <- harmoniseGWAS(obsgwas, minMAF=0.005, minINFO=0.4, beta_colname="EFFECT", se_colname="SE", snpID_colname="ID_dbSNP", EA_colname="ALT", NEA_colname="REF", EAfreq_colname="ALT_FREQ", BP_colname="BP", pvalue_colname="P", INFO_colname="INFO")
    See troubleshooting 2 if there are difficulties running this step without any errors.
    ***Note:*** In the arguments of the harmoniseGWAS function (above), specify the name of the column that contains each variable. The effect estimate column name is passed in the beta_colname argument, the standard error column name in se_colname, the variant name column name in snpID_colname, the effect allele column name in EA_colname, the non-effect allele column name in NEA_colname, the effect allele frequency column name in EAfreq_colname, the base-pair position column name in BP_colname, the p-value column name in pvalue_colname, and the imputation quality metric column name in INFO_colname. Specify the minimum MAF and minimum imputation quality metric level in minMAF and minINFO respectively. The defaults are minMAF=0.005, minINFO=0.4.
  b. The list object of GWAS data frames returned by harmoniseGWAS will have the column names “beta”, “SE”, “rsID”, and “EAF”, respectively for the effect estimates, their standard errors, the variant IDs, and the effect allele frequencies. These columns, in this name format, are needed to run the fine-mapping functions.
    **CRITICAL:** The variant IDs may take on any form, including combinations of rsIDs and position-based names, but missing values are not permitted and if a variant exists in multiple GWASs, it must have the same variant ID in those GWASs; they are the same IDs as those passed in the columns specified in the snpID_colnname argument.

### Calculation of latent factor GWAS summary statistics

**Timing: <5 min**

This step includes a factor analysis of the observed traits, the calculation of latent factor contributions to the observed traits, and the calculation of latent factor GWAS summary statistics. The contributions aid interpretation of the latent factors.

8. Open R and load the required packages, installing them first if necessary.

> library(flashfmZero)

> if (!requireNamespace("psych", quietly = TRUE)) {

> install.packages("psych")

> }

> library(psych)

9. Perform factor analysis of the observed traits.
  a. Select the number of latent factors for factor analysis using Horn’s parallel method, available in the “psych” R library^14^:
    > facheck <- psych::fa.parallel(covY, fm="ml", n.obs=N,fa="fa", plot=FALSE)
    ***Note:*** Above, covY is the trait covariance matrix, N is the median number of observed traits; fa=“fa”, fm=“ml” indicate maximum likelihood factor analysis. The number of latent factors is given in facheck$nfact.
  b. Perform factor analysis:
    > faY <- psych::fa(r=covY, nfactors=facheck$nfact, fm="ml", rotate="varimax")
10. Calculate latent factor contributions to the traits:

***Note:*** In the contributions (Contributions above) matrix, the observed traits (in rows) are ordered by the column index of their maximum contributing latent factor (in columns). In the factor loading matrix (FAloadings above) the observed traits (in rows) and the latent factors (in columns) have the same order as in the Contributions matrix.

> latent_terms <- flashfmZero::factor_contributions(faY$loadings)

> Contributions <- latent_terms[[1]]

> FAloadings <- latent_terms[[2]]

11. Calculate latent factor GWAS summary statistics:
  a. Re-order the trait covariance matrix (covY) and the list object containing GWAS summary statistics of the observed traits (obsgwas) to match the trait order of the factor loadings matrix:
    > covY <- covY[rownames(FAloadings),rownames(FAloadings)]
    > obsGWAS <- obsgwas[rownames(FAloadings)]
  b. Calculate latent factor GWAS summary statistics from the observed trait GWAS summary statistics and the observed trait covariance matrix.
    > latent_gwas <- flashfmZero::latentGWAS(obsGWAS = obsGWAS,
    covY = covY, L = FAloadings, beta_colname="beta", se_colname="SE", snpID_colname="rsID", EAF = obsGWAS[[1]]$EAF)
    ***Note:*** In this example we calculate summary statistics for 25 latent factors and 891,327 variants on chromosome 1.

### Latent factor fine-mapping

**Timing: <1 min**

In this step the genetic associations of each latent factor that has a signal in the region of interest are fine mapped. When multiple factors have a signal in the region, information common to the factors can be leveraged by applying flashfmZero. The output of each analysis includes a credible set (a short-list of potential causal variants) for each latent factor and each variant’s marginal posterior probability (MPP) of causal association with each latent factor.

12. Open R and load the required packages, installing if needed.

> library(flashfmZero)

> packages <- c("bigsnpr", "ggplot2")

> for (pkg in packages) {

> if (!requireNamespace(pkg, quietly = TRUE)) {

> install.packages(pkg)

> }

> library(pkg, character.only = TRUE)

}

13. Identify the latent factors with a genome-wide significant (p<5✕10^−8^) signal in the region, and if there are no such latent factors, then stop the analysis:

> check <- sapply(latent_gwas,function(x) any(x$p_value<5E-8))

> fm_traits_latent <- names(check[check==TRUE])

> if(length(fm_traits_latent)>0) { tmp1 <- latent_gwas[fm_traits_latent] }

> try(if(length(fm_traits_latent)==0) stop("No latent factor has a signal"))

14. Format the genotype files and calculate the linkage disequilibrium (LD) between variants in the region of interest.
  ***Note:*** In this example, we assume that the genotypes are stored in 8-bit encoded Oxford BGEN format.
  **CRITICAL:** If LD information is available in a correlation matrix (perhaps from another source) then ensure that the row and column names of the LD matrix are the variant IDs used in the GWAS summary statistics files and that the order of the variants used for the LD matrix is the same as the order of the variants in the summary statistics files, then skip steps a and b and move to step c.
  a. Obtain reference alleles at each variant by using qctool from the command line:
    $ qctool -g genotypes_chr1.bgen -snp-stats -osnp snp-stats-chr1.txt
    ***Note:*** The file snp-stats-chr1.txt contains variant-level summary statistics, including the reference and alternate alleles, to which the variants of the GWAS summary statistics need to be aligned.
  b. Load the genotype data, retaining only the variants that are in the region of interest.
    > snp_info <- read.table("./snp-stats-chr1.txt",header=TRUE)
    > list_snp_id <- list()
    > ind <- which(snp_info$bp >= 3441528 & snp_info$bp <= 3959487)
    > list_snp_id[[1]] = paste(snpinfo$chromosome[ind], snpinfo$position[ind], snpinfo$A_allele[ind],snpinfo$B_allele[ind], sep="_")
    > bigSNP_files = snp_readBGEN("./genotypes_chr1.bgen","∼/tmp", list_snp_id, read_as = "dosage")
    > bigSNP<-attach(bigSNP_files)
    ***Note:*** Here, we consider the *SMIM1* region on chromosome 1, with start and end physical coordinates of 3441528 and 3959487 respectively.
  c. Align the latent factor GWAS summary statistics to make them consistent with the alleles used to calculate LD:
    > RPinfo <- data.frame(chromosome=snpinfo$chr, rsID=paste(snpinfo$chr, snpinfo$pos, snpinfo$a1, snpinfo$a2, sep="_"), BP=snpinfo$pos, allele1=snpinfo$a1, allele2=snpinfo$a2)
    > sig_gwas_latent <- vector("list",length(fm_traits_latent))
    > for(i in 1:length(fm_traits_latent)) {
    > sig_gwas_latent[[i]] <- alignGWAS(tmp1[[i]],RPinfo)
    > }
    ***Note:*** It does not matter if a1 is the effect or non-effect allele, as this step is only to ensure that the summary statistics and LD are aligned.
  d. Hard call the genotypes using a call probability threshold of 0.2 and retain those variants with less than 20% missing data.
    > bg_genos <- apply(bigSNP$genotypes[,],2,LDqc)
    > keep_vars <- apply(!is.na(bg_genos),2,mean) >=0.8
    ***Note:*** In this region, 2,391 variants remain.
  e. Calculate a matrix of pairwise *r* statistics:
    > ld_matrix <- bigcor(bg_genos[,keep_vars], size=min(2000, sum(keep_vars)),fun="cor", use="p")
15. Re-name the variant IDs in the latent factor GWAS summary statistics and the LD matrix so that they start with a character and do not contain a “:”.

> corXnames <- paste0("chr",rownames(corX))

> rownames(corX) <- colnames(corX) <- corXnames

> sig_gwas_latent_names <- paste0("chr",sig_gwas_latent[[1]]$rsID)

> for(i in 1:length(sig_gwas_latent)) { sig_gwas_latent[[i]]$rsID <- sig_gwas_latent_names }

> names(sig_gwas_latent) <- fm_traits_latent

16. If there is only one latent factor with a genetic association signal in the region, then run JAMdwithGroups, and if multiple latent factors have a genetic association signal, then run FLASHFMZEROwithJAMd, which will output both single and multi-trait results.

> if(length(fm_traits_latent)==1){

> FM_latent <-JAMdwithGroups(sig_gwas_latent[[1]], N=N, corX, save.path="tmpDIR",r2.minmerge=0.8, cred=0.95)

> FM_latent_CS <- FM_latent$CS

> } else {

> FM_latent <-FLASHFMZEROwithJAMd(sig_gwas_latent, corX,

 N = N, save.path="tmpDIR", NCORES=length(sig_gwas_latent), r2.minmerge=0.8)

> FM_latent_CS <-allcredsetsPP(FM_latent$mpp.pp, cred=.95)

> names(FM_latent_CS$fm) <- fm_traits_latent

> names(FM_latent_CS$flashfm) <- fm_traits_latent

> }

***Note:*** In the above code, set N to the actual sample size of your GWASs. The cred argument specifies the credibility threshold for the construction of credible sets. The default value is cred=0.99 (specifying 99% credible sets), but here cred=0.95 is set to construct 95% credible sets.

**CRITICAL:** The flashfmZero library must be loaded into the R workspace to ensure that all Java dependencies are available.

If the code does not run to completion see troubleshooting 3.

17. A table summarizing the latent factor fine-mapping results may be constructed using the FMsummary_tablefunction included in the flashfmZero library:

> FMsummary_table(FM_latent, traitnames = fm_traits_latent, cred=0.95)

***Note:*** Again, the cred argument specifies the credibility threshold for the construction of credible sets and has a default value of 0.99. In this example we set cred=0.95 to construct 95% credible sets.

***Note:*** The output of this function for the *SMIM1* region is presented in Table 1. Its format is described in the expected outcomes section.

**Figure 1:**
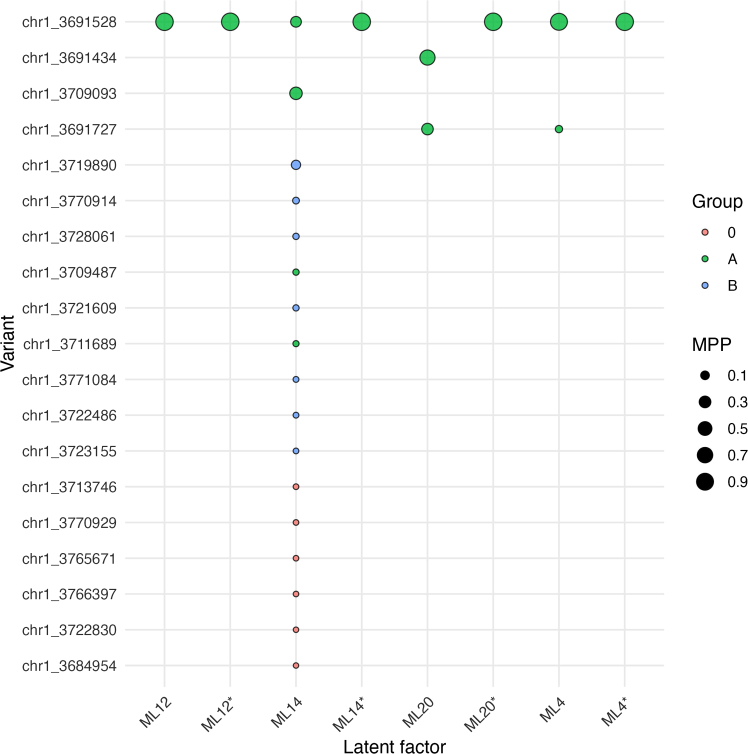
Summary of 95% credible sets (CS95) from the flashfmZero fine mapping of associations with latent factors in the *SMIM1* region The rows correspond to variants belonging to at least one CS95, indicated by the variant ID labels on the *y*-axis. The columns correspond to the CS95s from the fine-mapping analyses indicated by the labels on the *x*-axis. The univariate latent factor CS95s are denoted by the latent factor name (e.g., ML12), while the multi-trait latent factors CS95s are identified by appending an asterisk to the name of a contributing latent factor (e.g., ML12∗). The presence of a filled circle indicates that the corresponding variant is a member of the corresponding CS95. The fill colors (labelled by letters in the legend) indicate a group of high-LD variants (with MPP > 0.01, *r*^*2*^ > 0.8) derived the fine-mapping method; the salmon circles (labelled “0”) correspond to variants with MPP<0.01 and which were therefore not assigned to a group. The area of each circle is proportional to the marginal posterior probability (MPP) that the variant is causally associated with the trait. The multi-trait latent factor CS95s each contain one variant, refining the univariate latent factor CS95s.

**Figure 2:**
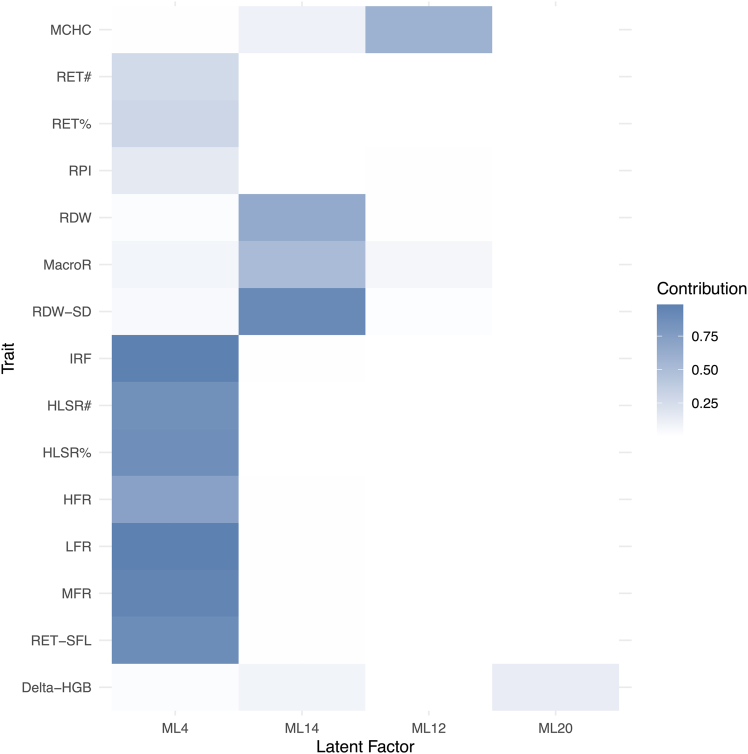
Interpretation of the latent factors with signals in *SMIM1* The contributions of latent factors to the observed traits are displayed to facilitate interpretation, where those contributions are at least 10%. A darker shade of blue indicates a greater contribution. The block structure indicates that the same latent factors act as primary contributors to groups of traits.

18. The credible sets from single and joint latent factor fine-mapping can be summarized in a bubble plot, as illustrated in Figure 1.
  a. Format lists of credible sets output by flashfmZero into a long data.frame that includes the SNP group labels of each variant.
    ***Note:*** Variants that were not assigned a SNP group are labelled as group “0”. The labels of credible sets for latent factors are given by the latent factor name appended with an asterisk.
    > long_df_fm <- bind_rows(FM_latent_CS$fm, .id = "source")
    rownames(long_df_fm) <- NULL
    > long_df_fm$Group <- groupIDs.fn(FM_latent$snpGroups[[1]], long_df_fm$SNP)
    > long_df_flashfm <- bind_rows(FM_latent_CS$flashfm, .id = "source")
    > rownames(long_df_flashfm) <- NULL
    > long_df_flashfm$source <- paste0(long_df_flashfm$source,"∗")
    > long_df_flashfm$Group <- groupIDs.fn(FM_latent$snpGroups[[2]], long_df_flashfm$SNP)
    > df <- rbind(long_df_fm,long_df_flashfm)
    > ind <- which(nchar(df$Group)>1)
    > df[ind,"Group"] <- "0"
    > names(df)[1] <- "trait"
    > df <- df %>%
     group_by(SNP) %>%
     mutate(maxMPP = max(MPP)) %>%
     ungroup() %>%
     mutate(SNP = reorder(SNP, maxMPP))
  b. Construct bubble plots of the variants in the credible sets output by flashfmZero.
    > ggplot(df, aes(x = trait, y = SNP, size = MPP, fill = Group)) +
     geom_point(shape = 21, alpha = 0.8, color = "black") +
     scale_size(range = c(1, 5),
     breaks = c(0.1, 0.3, 0.5, 0.7, 0.9), limits = c(0, 1), name = "MPP") + guides(size = guide_legend(
    override.aes = list(shape = 21, fill = "black", color = "black", alpha = 1))) + theme_minimal(base_size = 12) +
     theme(axis.text.x = element_text(angle = 45, hjust = 1),
     panel.grid.major = element_line(color = "gray90"),
     panel.grid.minor = element_blank(),
     plot.title = element_blank()) +
     labs(x = "Latent factor", y = "Variant", fill = "Group")
19. Plot the latent factor contributions to the observed traits to facilitate the interpretation of the results.
  a. Filter, retaining only the traits that have a contribution of at least 10% from the latent factors that were fine-mapped and convert to a long-form data.frame, preserving row/column order.
    > mat <- Contributions[,fm_traits_latent]
    > mat <- as.matrix(mat[apply(mat, 1, function(x) any(x > 0.1)), ])
    > df <- as.data.frame(as.table(mat))
    > colnames(df) <- c("Trait", "LF", "Contribution")
    > df$Trait <- factor(df$Trait, levels = rownames(mat))
    > df$LF <- factor(df$LF, levels = colnames(mat))
  b. Construct a heat map displaying the contributions of the latent factors to the observed traits.
    > ggplot(df, aes(x = LF, y = Trait, fill = Contribution)) +
     geom_tile() + labs(x="Latent Factor") +
     scale_fill_gradient(low = "white", high = "steelblue") + theme_minimal()
    > save("Contributions.pdf")
    ***Note:*** This plot is displayed in Figure 2. It indicates that ML20 primarily contributes to Delta-HGB, ML12 to MCHC, ML14 to RDW-SD, MacroR, and RDW, whereas ML4 is the primary contributor to RET-SFL, MFR, LFR, HFR, HLSR%, HLSR#, and IRF.

**Table 1:** Output of the FMsummary_table function from the fine-mapping of associations with latent factors in the *SMIM1* region

Approach	Trait	99% credible set size (CS99 size)	Name of SNP with the top MPP	MPP	SNP group containing the top MPP	Size (number of variants in group) of this SNP group	MPPg	Variants with MPP>0.90	MPP of variants with MPP>0.90	SNP groups (MPPg > 0.90)	Size of SNP groups (MPPg > 0.90)	MPPg of groups with MPPg>0.90
JAM-latent-factor	ML12	1	chr1_3691528	0.993	A_lat	6	1.000	chr1_3691528	0.993	A	6	1.000
JAM-latent-factor	ML14	37	chr1_3709093	0.378	A_lat	6	0.654	NA	NA	NA	0	NA
JAM-latent-factor	ML20	4	chr1_3691434	0.677	A_lat	6	0.987	NA	NA	A	6	0.987
JAM-latent-factor	ML4	5	chr1_3691528	0.920	A_lat	6	0.977	chr1_3691528	0.920	A	6	0.977
flashfm-latent-factor	ML12	1	chr1_3691528	1.000	A_lat	1	1.000	chr1_3691528	1.000	A	1	1.000
flashfm-latent-factor	ML14	1	chr1_3691528	1.000	A_lat	1	1.000	chr1_3691528	1.000	A	1	1.000
flashfm-latent-factor	ML20	1	chr1_3691528	1.000	A_lat	1	1.000	chr1_3691528	1.000	A	1	1.000
flashfm-latent-factor	ML4	1	chr1_3691528	1.000	A_lat	1	1.000	chr1_3691528	1.000	A	1	1.000

Note that the table column names returned by the function are “Approach”, “Trait”, “CS99size”, “topSNP”, “topMPP”, “topGroup”, “topGroupSize”, “topGroupMPP”, “SNP_gt90”, “MPP_gt90”, “group_gt90”, “group_gt90_size”, “MPPg_gt90”, which have been defined here.

## Expected outcomes

For each latent factor, the FMsummary_table function will output one row for single latent factor fine-mapping and one row for joint latent factor fine-mapping. The FMsummary_table output from the example of fine-mapping of the *SMIM1* region is displayed in Table 1. The output includes the number of variants in each credible set, the variant with the highest MPP (and the value of the MPP), and a list of variants with MPP>0.90. The output also indicates SNP groups, which are groups of SNPs with non-negligible MPP that can be viewed as interchangeable for the purposes of fine-mapping given that they are in high LD (*r*^*2*^>0.80); SNP groups depend on both the LD structure and the fine-mapping results, so groups derived from joint latent factor fine-mapping tend to be smaller than those derived from single latent factor fine-mapping.

## Quantification and statistical analysis

Fine-mapping results are output for each latent factor, including a credible set and variant MPPs. A typical threshold for selecting high-confidence variants is MPP>0.90. The fine-mapping functions also output SNP group-level results, e.g., MPPg, the marginal posterior probability of causality for variants belonging to a particular SNP group. These SNP groups represent distinct signals in the region.

## Limitations

This protocol has been designed for quantitative traits that have been measured in samples from a single genetic ancestry group. LD should be calculated from in-sample genotype data or from a sample that has similar genetic ancestry. The GWAS summary statistics files and the LD file must be aligned to the same alleles. The calculation of latent factor GWAS summary statistics is only possible at variants that are present in all observed traits GWAS.

## Troubleshooting

### Problem 1

The flashfmZero library cannot be installed.

flashfmZero could be installed with ease on versions of R > 4.2.1 and is compatible with all platforms (Linux, Microsoft Windows, macOS). All details on installation are also provided at https://github.com/jennasimit/flashfmZero.

### Potential solution

• All platforms: ensure that a Java JDK is installed, as described at https://docs.oracle.com/en/java/javase/24/install/overview-jdk-installation.html.
• Microsoft Windows platform: ensure that Rtools (https://cran.r-project.org/bin/windows/Rtools/) is installed.
• macOS platform: ensure that Xcode (free on the Apple App Store) and a Fortran compiler are installed. R 4.3.0 and higher use universal GNU Fortran 12.2 compiler and an installer package, as available here: https://mac.r-project.org/tools/gfortran-12.2-universal.pkg.

### Problem 2

An error occurs when harmonizing the GWAS data using the harmoniseGWAS function.

### Potential solution

Ensure that all GWAS files have the same column names. It is fine if some files have extra columns, but they all must contain the column names specified by the user in the harmoniseGWAS arguments.

### Problem 3

The fine-mapping code does not run to completion.

### Potential solution

There are two possible solutions to this:

• Ensure that all the variant IDs begin with a character and do not contain any colons (“:”), “<”, or “>”.
• Ensure that the variant IDs in the GWAS match the variant IDs in the LD matrix.

The variant IDs can take any form that satisfies these requirements.

## Resource availability

### Lead contact

Further information and requests for resources should be directed to and will be fulfilled by the lead contact, Jennifer Asimit (jennifer.asimit@mrc-bsu.cam.ac.uk).

### Technical contact

Technical questions on executing this protocol should be directed to and will be answered by the technical contact, Jennifer Asimit (jennifer.asimit@mrc-bsu.cam.ac.uk).

### Materials availability

This study did not generate new unique reagents.

### Data and code availability

Source data for the examples in this paper and an R script for its analysis are available at https://github.com/jennasimit/flashfmZero-example-data and https://doi.org/10.5281/zenodo.15785324.^15^ Software for flashfmZero^1^ is available at https://jennasimit.github.io/flashfmZero/ and on Zenodo: https://doi.org/10.5281/zenodo.13305579.^2^

## Acknowledgments

J.L.A. is supported by the UK Medical Research Council (MR/R021368/1, MC_UU_0004/1), the Isaac Newton Trust, and Medical Research Foundation (MRF-DA-111). W.J.A. is supported by NHS Blood and Transplant. A.S.B. was supported by core funding from the British Heart Foundation (RG/18/13/33946: RG/F/23/110103), the NIHR Cambridge Biomedical Research Centre (NIHR203312) [∗], the BHF Chair Award (CH/12/2/29428), and by Health Data Research UK, which is funded by the UK Medical Research Council, Engineering and Physical Sciences Research Council, Economic and Social Research Council, Department of Health and Social Care (England), Chief Scientist Office of the Scottish Government Health and Social Care Directorates, Health and Social Care Research and Development Division (Welsh Government), Public Health Agency (Northern Ireland), British Heart Foundation, and the Wellcome Trust.

Participants in the INTERVAL randomized controlled trial were recruited with the active collaboration of NHS Blood and Transplant England (www.nhsbt.nhs.uk), which supported fieldwork and other elements of the trial. DNA extraction and genotyping were co-funded by the National Institute for Health and Care Research (NIHR), the NIHR BioResource (http://bioresource.nihr.ac.uk), and the NIHR Cambridge Biomedical Research Centre (BRC-1215-20014) [∗]. The academic coordinating center for INTERVAL was supported by core funding from the NIHR Blood and Transplant Research Unit (BTRU) in Donor Health and Genomics (NIHR BTRU-2014-10024), NIHR BTRU in Donor Health and Behaviour (NIHR203337), UK Medical Research Council (MR/L003120/1), British Heart Foundation (SP/09/002; RG/13/13/30194; RG/18/13/33946), NIHR Cambridge BRC (BRC-1215-20014; NIHR203312) [∗], and by Health Data Research UK. A complete list of the investigators and contributors to the INTERVAL trial is provided in the study by Moore et al.^16^ The academic coordinating center would like to thank blood donor center staff and blood donors for participating in the INTERVAL trial.

We thank Parsa Akbari for making available blood cell traits from the INTERVAL study adjusted for technical variation.

For the purpose of Open Access, the authors have applied a CC BY public copyright license to any Author Accepted Manuscript version arising from this submission.

∗The views expressed are those of the authors and not necessarily those of the NIHR or the Department of Health and Social Care.

## Author contributions

J.L.A. conceptualized the study and developed the statistical methodology and related software. W.J.A. prepared and analyzed the data. A.S.B. provided the data. W.J.A. and J.L.A. wrote the first draft of the paper. W.J.A., A.S.B., and J.L.A. reviewed and approved the paper.

## Declaration of interests

A.S.B. reports institutional grants outside of this work from AstraZeneca, Bayer, Biogen, BioMarin, Bioverativ, Novartis, Regeneron, and Sanofi.
